# Reconceptualising the commercial determinants of health: bringing institutions in

**DOI:** 10.1136/bmjgh-2023-013698

**Published:** 2023-11-27

**Authors:** Rob Ralston, Charlotte Godziewski, Eleanor Brooks

**Affiliations:** 1 Global Health Policy Unit, School of Social & Political Science, The University of Edinburgh, Edinburgh, UK; 2 SPECTRUM Consortium (Shaping Public Health Policies to Reduce Inequalities and Harm), London, UK; 3 Department of International Politics, City, University of London, London, UK

**Keywords:** health policies and all other topics, health policy, public health

## Abstract

The concept of the ‘commercial determinants of health’ (CDOH) has been developed by public health researchers as a way to describe the political economy of corporations and the impact of their practices on health, social inequalities and climate change. In this analysis, we assess the conceptual work that has developed this field and the influence of the more established ‘social determinants of health’ models. We highlight the dominance of epidemiologic and biomedical concepts on understandings of structure and agency in the CDOH literature and argue that the terminology of ‘risk factors’, ‘drivers’ and ‘pathways’ reflects an agent-centred approach. We suggest that, as a result, there is a tendency to overlook the importance of political institutions in shaping the exercise of corporate power. Our analysis seeks to ‘bring institutions in’ to CDOH research, using the empirical cases of Health in All Policies and Better Regulation in the European Union to highlight how institutional contexts shape political legitimacy and accountability, and in turn the strategies of corporate actors. Institutionalist approaches, we argue, have the potential to develop and expand understandings of CDOH by opening the black box between agency and structure.

**SUMMARY BOX**

- The commercial determinants of health (CDOH) is a rapidly developing area of interest in public health. Most of this work is agent-centred; it looks at the behaviours and strategies of corporate actors and situates them within macrolevel structures of globalising (neoliberal) capitalism.
- The role of institutions, so far, has been a blind spot in the CDOH literature—and whilst some research engages with institutions, it tends to consider them as the space for corporate agency, rather than the object of study itself.
- Our analysis demonstrates how institutionalist theories can expand the understanding of how the CDOH operate. Applying such theories connects microlevel agency with macrolevel structures. This enables public health researchers to better differentiate between modalities of power, and explain either how institutions empower commercial actors or how institutions themselves become CDOH.
- Institutionalist theories are underexplored in the CDOH literature and have analytical value in explaining how corporate power affects public health.

## Introduction

The concept of the ‘commercial determinants of health’ (CDOH) has been developed by public health researchers as a way to describe the political economy of corporations and the impact of their practices on health, social inequalities and climate change. While the political and economic power of industries (such as tobacco, processed food, alcohol, plastic and fossil fuels) is well-established in public health research, CDOH is an umbrella term that packages it for other academic disciplines and policy audiences. In this analysis, we assess the conceptual work that has developed this field and the influence of the more established ‘social determinants of health’ models. We highlight the influence of epidemiologic and biomedical concepts on understandings of structure and agency in the CDOH literature and argue that the terminology of ‘risk factors’, ‘drivers’ and ‘pathways’ reflects an agent-centred approach. We suggest that, while advances have been made—notably in the recent Lancet Series on the Commercial Determinants of Health—there remains a tendency to overlook the importance of *institutions* in shaping the structural and instrumental power of corporate actors. This analysis seeks to ‘bring institutions in’ to CDOH research, using the empirical cases of Health in All Policies (HiAP) and Better Regulation in the European Union (EU) to highlight how institutional contexts shape political legitimacy and accountability, and in turn the strategies of corporate actors. We argue that taking an institutionalist approach has the potential to develop and expand understandings of CDOH.

## Agency and structure in the commercial determinants literature

Public health research on CDOH has proliferated over the past decade, with a corresponding increase in analytical frameworks of the relationship between corporate practices and planetary health. While these frameworks vary in scope, most tend to conceptualise the relationship between commercial actors and health with implicit (and often explicit) reference to biomedical and social epidemiologic frameworks, adopting many of the theories, models and terminology developed in the more established literature on the ‘social determinants of health’. For example, conceptual work on CDOH draws on the terminology of ‘proximal’ (or downstream) individual lifestyle behaviours (eg, smoking, alcohol and processed food consumption, physical activity) as significantly shaped by ‘distal’ (or upstream) social and political contexts. This conceptualisation of the relationship between health and socioeconomic factors has formed the basis of most CDOH frameworks, in which commercial activities are understood as pathways that link distal and proximal determinants.^1^ These frameworks detail how corporations impact health across multiple scales and social contexts, from the distribution, pricing and promotion of brands and products, which nudge and shape individual behaviours at the microlevel, to macrolevel paradigms that affect how markets and corporations are regulated.

CDOH frameworks often focus on the agency of commercial actors and, in particular, how they exert disproportionate influence over regulatory politics and public policy-making.^2 3^ Drawing on systematic research of the tobacco industry, these frameworks have concentrated on the exercise of corporate power through political lobbying, marketing, promoting self-regulation and multistakeholder governance, corporate social responsibility initiatives, funding scientific research, and influencing political and public discourse of policy issues. For example, Kickbusch *et al* conceptualise CDOH as ‘strategies and approaches used by the private sector to promote products and choices that are detrimental to health’.^4^ This agent-centred approach is visible in other widely cited definitions, such as Knai *et al*s’ definition of CDOH as the ‘adverse health impacts attributable to commercial activities and strategies employed by unhealthy commodity industries (UCIs) to promote products which damage health’.^5^ These definitions are illustrative of a tendency within CDOH frameworks to centre on the agency of corporate actors and their ability to influence regulatory and political systems.

Agent-centered approaches have been complemented by a shift toward analysing forms of structural power.^6–8^ The latter refers to the macrolevel economic and political systems of capitalism that shape the strategies of corporations, and how these systems are themselves shaped through corporate power. Wood *et al*, for instance, describe ‘underlying contexts, dynamics, paradigms’ as being shaped by corporate power, describing how neoliberalism, market fundamentalism, and the internationalisation of trade and finance, have created regulatory settings amenable to maximising production and profit.^8^ In so doing, the authors draw—as many others do—on theories of power.^9 10^ The most influential framework is Lukes’ (1975; 2005) *three faces of power* (decision-making, non-decision-making and ideological),^11^ which also informs other prominent models cited in the CDOH literature, notably, Doris Fuchs and Lederer’s three-dimensional model of instrumental, discursive and structural power.^12^ These theories have been operationalised in ways that highlight agency and structural power, in which corporate actors exercise disproportionate influence within political and economic systems. A great deal of the CDOH literature^6 7 10 13^ has thus tended to emphasise the agency that corporate actors exercise by referencing strategic action, and/or structural power explanations; these highlight the role of capitalist systems and neoliberal logics, where agency and structure are analytically distinct but difficult to disentangle in practice.^14^ Taking inspiration from contemporary political economy and political science literatures, we consider institutional and ideational contexts as mediating the structural and instrument power of business actors.^15^

While the relationship between structure and agency is an enduring question in political science, the focus on macro structures and micro strategy has arguably led to a neglect of *institutions*, which remain a black box in understandings of CDOH despite a focus on institutional contexts in the wider business power literature.^16 17^ Recent work has begun to address this imbalance. For example, Gilmore *et al* emphasise the agency exerted by commercial actors in promoting multistakeholder initiatives, noting that this strategy has been ‘so successfully established that many institutions, including UN bodies and governments, have shifted towards working with commercial actors even within the health arena’.^3^ However, as the previous example illustrates, there remains a tendency to privilege agency in imposing or co-opting particular institutional forms. Put differently, institutions continue to be viewed in agent-centred terms, as the arenas in and over which corporate actors exert influence. This is arguably reflected in the focus of the CDOH literature on regulatory capture: a concept that describes how regulatory policy is strategically directed away from the public interest and towards the interests of the regulated industry,^18^ emphasising the agency of corporate actors in imposing their preferred policy agendas.^7 9^ We argue that this focus on agency has led to a neglect of political institutions as an object of theorisation and a determining factor in shaping the exercise of corporate power. To be clear, we do not intend to downplay how agency is exercised to define and shape debates over health and environmental governance, or to minimise the extent to which corporate actors exercise disproportionate influence over politics and policy. Rather, our point is that concentrating on agency only tells part of the story and that a more holistic approach shows that strategic action occurs in institutional settings that have their own history, context and development, and are shaped by a variety of actors, interests and ideas. In other words, we argue that the institutional contexts and dynamics that shape the political economy of health are not reducible to regulatory capture alone, but instead require treating institutions as a distinct object of study.

Despite recognition of the importance of institutions within critical public health,^19 20^ institutionalist theories are surprisingly absent in conceptual work on CDOH. To address this gap, we engage with the political science literature,^21^ demonstrating how institutionalist theories—including but, crucially, moving beyond rational choice approaches—can shed new light on the relationship between corporate actors and the political economy of health. Drawing on the empirical cases of HiAP and Better Regulation within the EU, we highlight how institutional environments shape what is perceived as legitimate, possible and desirable, and in turn the strategic action of corporate actors.

## Bringing institutions into commercial determinants research

Institutionalist approaches are firmly established within the political sciences as a means of understanding policy change. The term ‘new institutionalism’ is used to refer to three distinct accounts of institutions: historical, rational choice and sociological, with the later addition of discursive institutionalism as a fourth variant that is concerned with how ideas and discourse shape (and are shaped by) institutions.^22^ While there are important differences between institutionalist approaches in terms of how they conceptualise the relationship between structure and agency, they share a focus on institutions as relatively stable and enduring sets of rules, norms and practices, whether formal (written) or informal (unwritten), hard (creating obligations) or soft (voluntary).^23 24^ The formal rules, compliance procedures and operating practices of institutions prescribe appropriate behaviour, empower and constrain actors (differently), and shape resource and capability distribution.^25^

Institutions play two main roles in (health) policy: first, they affect the degree of power that actors have over decision-making and its outcomes and, second, the institutional position of actors influences the definition of their interests, responsibilities and relationships.^26^ Peter Hall argues that institutions can structure ‘the basic logic of political rationality for many actors by altering their relationship to other actors’.^26^ This is particularly relevant for understanding corporate power, where actors move within and between different levels of political systems and develop strategies to take advantage of different institutional opportunities.^27^ The regulatory politics of obesity policy offers an example of how institutions shape power relations between actors and the formation of corporate political strategies. Governance tools like multistakeholder partnerships with the food industry affect the power that corporate actors have over decision-making in relation to other actors, while the interests and objectives of those actors are defined by their institutional position.^28 29^ The power and position that corporate actors have within such institutional settings is shaped by the setting itself and its underlying logics. In other words, these actors can occupy privileged positions and have decision-making power, which does not necessarily relate to their agency, but is rather mediated by institutions. Moreover, institutions often exhibit ‘path dependency’, meaning that decisions made when an institution (or policy) is created shape possible/feasible future decisions and make reversal of decisions difficult.^30^ Institutionalist approaches are analytically distinct from perspectives within the current CDOH literature to the extent that they problematise the relationship between context, agency and structure, creating space to consider institutional formation, transformation and how this affects strategic action. In order to illustrate, we present two empirical examples of how institutional contexts (and the ideas, rules and procedures that constitute them) shape the opportunities and strategic action of corporate actors.

## ‘Health in all policies’

Using a (discursive) institutionalist lens to analyse the introduction of HiAP within the EU institutions, and how this concept became interpreted as inherently about multistakeholder engagement, provides useful insights into the politics of defining HiAP, the interplay between institutional and ideational forms of power, and how this tends to benefit corporate actors.

HiAP is a broad policy agenda intended to mainstream health considerations across all policy areas. It stems from the recognition that the most crucial determinants of health are social, political and/or economic, and not governed through health policies.^31^ While HiAP is associated with various principles, such as the promotion of intersectoral coordination to improve policy coherence for health, it lacks a single, clear definition. This is in part strategic, as maintaining a level of vagueness has been instrumental to promote the concept within policy spaces, notably in the EU under the 2006 Finnish Presidency.^32^ The definition and framing of HiAP is, hence, a site of discursive power struggle and inherently political.^33^

When the concept was introduced in the European Commission, it became (re)defined as a tool of multistakeholder governance: the idea that HiAP required the involvement of all sectors of society, including ‘NGOs, industry, academia and the media’ was presented as obvious. This definition was criticised by the Finnish HiAP advocates, who were seeking to promote HiAP as a cross-policy sector collaboration.^32 34^ HiAP does not intrinsically entail a multistakeholder meaning.^35^ Rather, the adoption of this definition in the case of HiAP in the EU is a product of agential, ideational and institutional power dynamics. The definition benefits corporate actors and has been promoted by them: by blurring the lines between the governing and the governed,^36^ multistakeholder approaches allow corporate actors to institutionalise their position as indispensable decision-makers, and to diffuse responsibility for public health across all levels of society, including back onto individuals.^28^

However, the success of the multistakeholder framing of HiAP should not be reduced to corporate actor’s strategic action alone. The resilience of multistakeholderism illustrated here also lies in the ideational power it has acquired, as well as the nature of the formal EU institutional context, in particular that of supranational bodies like the Commission. Ideational power means that multistakeholderism has become a deeply established norm within governing institutions, to the point that it is taken for granted and provides the frame for how HiAP is interpreted within the EU institutions. Getting multistakeholderism to this status is, of course, not independent of (prior) instrumental agency.^37^ Nevertheless, the result is that commercial actors arguably no longer need to rely on such instrumental efforts to promote their inclusion at the decision-making table, because the idea of multistakeholder governance has become powerful enough to sustain itself. The *ideational* power of multistakeholderism also needs to be understood within the *formal* institutional constraints related to the EU’s limited conferred competencies in health, and the affinity of supranational EU institutions like the Commission towards ‘new modes of governance’ as a way to respond to perceptions of democratic deficit.^38^ These institutional factors which did not result from corporate strategy, though both converge in promoting a multistakeholder framing of HiAP.

## Better regulation

Introduced in the late 1990s and amended on numerous occasions since, the EU’s Better Regulation agenda seeks to improve the quality of EU legislation by strengthening its evidence base, increasing participation in policy-making and reducing the burden of legislation for businesses and citizens.^39 40^ In contrast to HiAP, the instruments of Better Regulation are pervasive, obligatory and deeply institutionalised within EU policy-making. They are embodied in a collection of formal and informal institutions—rules, norms, organisations, outputs—that is constantly evolving. In practice, the EU seeks to achieve ‘better’ regulation by requiring, for example, that all new pieces of legislation are preceded by an evaluation of existing policy in the same area, that all legislative initiatives are accompanied by impact assessments and that stakeholders are consulted at multiple points during the policy process. Better Regulation also sets the overarching objectives—beyond those that the policy itself is trying to achieve—that all policies must serve. These include not only the principles of subsidiarity and proportionality, but also those of burden reduction, transparency, participation, evidence-based policy-making and cost-effectiveness.

An institutional approach highlights the relevance of Better Regulation’s origins in the deregulatory, liberalising policies of the Thatcher and Reagan administrations in the UK and the USA, respectively, and how these origins are reflected in the rules and norms of the contemporary agenda. The institutional setting of Better Regulation has historically been one of economic competitiveness and growth; much as the logics of capitalism and neoliberalism constrain pursuit of health equity,^41^ so the logics of simplification and administrative burden shape the construction of regulation as ‘red tape’. A good example of the path dependence created by these past decisions and framings is the adoption of the new ‘one-in-one-out’ (OIOO) programme, which seeks to ‘(offset) newly introduced burdens […] by removing equivalent burdens in the same policy area’.^42^ Though resisted by the Commission for many years, OIOO now systematises ‘burden reduction’ within the policy process, reflecting the agenda’s institutional setting: ‘Offsetting burdens with one-in-one-out is acceptable if you think that the main problem is one of administrative obligations and costs. If you instead direct better regulation towards net benefits, the role of one-in-one-out has to be majorly discounted’.^43^

An institutional lens can also be used to consider the power that Better Regulation gives to specific actors, and how it structures actors’ relationships and interests. In setting the ‘rules of the game’ via provisions on consultation and input into the policy process, Better Regulation empowers those with capacity to engage, sets the parameters of that engagement and fosters participants’ interest in the way that the rules of the game are designed. It is for this reason, the tobacco and other industries were instrumental in influencing the design of Better Regulation and advocating for its implementation.^44^ Research has shown that the same actors that exercised instrumental power in shaping the agenda have since made discursive use of Better Regulation to argue against specific policies, such as the EU Tobacco Products Directive and the WHO Framework Convention on Tobacco Control.^28 45^ Japan Tobacco International, for instance, cites Better Regulation principles when asserting its ‘right and obligation’ to participate in policy-making in the EU and elsewhere.^46^ A similar logic underpins the development of the ‘innovation principle’, a device advocated by commercial actors to challenge the long-standing ‘precautionary principle’ of EU law, by institutionalising a requirement to consider ‘impact on innovation’ in all EU policy processes.^47^ Businesses and governments also played a central role in advocating for the introduction of OIOO. The temporal patterns of engagement in these examples might be observed within an agent-centred analysis but, as Hall notes, (historical) institutionalist analysis—and particularly concepts like path dependence—allows us to understand what defines and determines actors’ degree of power.^26^ By exercising instrumental power and lobbying higher level institutions at time ‘t’, commercial actors are able to establish and embed an institutional structure which benefits them—and reduces or negates the need for further instrumental efforts—at time t+1.

## What institutionalist approaches offer to the commercial determinants literature

The past decade has seen an increasing interest within public health research in integrating theories developed within the political sciences, with several important contributions on how institutions shape health policy.^19 32 48 49^ The CDOH literature is already making efforts to build this bridge, as its engagement with concepts of capitalism, neoliberalism and globalisation demonstrate, but it continues, for the most part, to link actors to (macro) systems without examining the processes, practices, norms and rules that shape and facilitate this link. Building on this, we argue that institutionalist approaches bring a concreteness and specificity to the study of the CDOH,^50^ with the potential to open the black box between corporate agency and (macrosystem) structure. Institutions are not a separate, blank landscape on which lobbyists exert ‘their’ (pre-existing) power. Rather, power—including of corporate actors—is better seen as relational and positional, and institutions are determining constructs which shape those relations and positions.

Institutions cannot be separated from agency, as indeed institutions are constructed through agents. As such, we do not wish to neglect or downplay the role of instrumental and strategic action by corporate actors seeking to influence institutions. However, agents are also constructed through institutions, which have their own contexts and history. Understanding both sides of that coin means going beyond treating institutions as passive objects shaped or ‘captured’ by an active corporate agent. It enables public health researchers to better differentiate between modalities of power: where does industry use instrumental power, and why does it often not need to? In this sense, institutionalist traditions can fine-tune the analysis of power dynamics at play in the CDOH, either by explaining how institutions empower commercial actors, or how institutions themselves become CDOH.

## Data Availability

All data relevant to the study are included in the article.
